# Dysphagia secondary to dermatomyositis treated successfully with intravenous immunoglobulin: a case report

**DOI:** 10.1186/1755-7682-1-12

**Published:** 2008-07-23

**Authors:** Deepak Joshi, Rizwan Mahmood, Peter Williams, Paul Kitchen

**Affiliations:** 1 Department of Gastroenterology, Medway Maritime Hospital, Kent, UK; 2 Department of Rheumatology, Medway Maritime Hospital, Kent, UK

## Abstract

A 46 year old woman presented with a one month history of rash and mylagia. The history, clinical findings and blood tests all supported a diagnosis of dermatomyositis. The patient later developed dysphagia and was successfully treated with intravenous immunoglobulin. Investigations and treatment of dysphagia in the context of dermatomyositis are discussed.

## Background

Dermatomyositis commomly presents with fatigue, proximal weakness, muscle tenderness and a characteristic rash. It can occur in conjunction with an underlying malignancy. Dysphagia can also occur and is associated with a 31% one year mortality [1]. The authors present a case of dermatomyositis in a 46 year old woman who developed dysphagia and was successfully treated with intravenous immunoglobulin.

## Case report

A 46 year old caucasian female presented with a one month history of a rash on the face and hands. She had also noticed generalised myalgia. There was no history of dysphagia, weight loss or night sweats. There had been no recent foreign travel. A past medical history of pernicious anaemia, treated with hydroxycobalamin injections, was noted. No other regular medications were being taken or had been started in the last month. Cardio-respiratory and abdominal examination was unremarkable. Power in all four limbs was graded MRC 5/5. A helitropic rash affecting both upper eyelids and a scaly violaceous eruption on the knuckles was noted. The full blood count, renal function and coagulation studies were normal. Serum B12 and folate levels were also normal. Serum creatine kinase (CK) was 9831 U/L (NR 20–180) and ESR was 54 mm/h (NR 0–20).

The history, clinical findings and blood tests all supported a diagnosis of dermatomyositis and therefore high dose prednisolone (60 mg OD) and methotrexate 10 mg per week with folic acid supplementation was commenced. The patient underwent a deltoid muscle biopsy which demonstrated patchy atrophy of fibres and inflammation around the fasicles compatible with dermatomyositis. Computed tomography of the abdomen, chest and pelvis was normal. Breast mammography revealed a normal parenchymal pattern with no features supportive of malignancy. Auto-antibodies including ANA and anti Jo 1 were negative. The patient was discharged 12 days later with significant improvement in symptoms. The CK continued to normalise 1888 U/L.

The patient was readmitted 21 days later with worsening dysphagia. Upper gastro-intestinal endoscopy demonstrated poor primary contractions with swallowing. Fibro-optic endoscopic evaluation of swallowing highlighted velo-pharyngeal dysfunction, inadequate clearance of bolus from the pharynx and evidence of residue in the valleculae and piriform fossa. Oesophageal and gastric transit studies confirmed oesophageal dysmotility affecting the medial and lower third of the oesophagus and mild gastric stasis. The prednisolone was increased to 60 mg. Given the findings of oesophageal and gastric delay, Intravenous immunoglobulin (IV IG) 40 g OD for three days was given, with a significant improvement in symptoms. Further IV IG was deemed unnecessary due to a paucity of symptoms. The patient was continued on prednisolone 60 mg for a further four weeks before being reduced and the methotrexate was increased to 15 mg per week. The patient continued to be followed up by both the gastroenterologists and rheumatologists. One year after her initial presentation, the patient described no dysphagia. Repeat fibro-optic endoscopy and oesophageal transit studies were not repeated given the resolution of symptoms.

## Discussion

The idiopathic inflammatory myopathies are divided into four subgroups: dermatomyositis (DM), polymyositis (PM), inclusion body myositis (IBM) and an overlap syndrome with mixed characteristics [2]. DM is a complement mediated microangiopathy affecting the skin and muscles [3]. Endocapillary deposition of the complement C5b-9 membraneolytic attack complex leads to the loss of capillaries, ischaemia and necrosis of muscle fibres [4]. The characteristic sign associated with DM is a heliotrope (blue-purple) rash of the upper eyelids. A flat, red rash on the face (often associated with periorbital oedema) and upper trunk is also often noted. In the hands, Gottron's papules (raised violaceous scaly eruption on the knuckles) and periungal erythema may be found. DM is associated with an occult malignancy in 20–30% of cases especially colorectal, bladder, lung, non-Hodgkin's lymphoma, ovarian, pancreatic and stomach cancers. The risk for developing a cancer remains for up to five years [5]. A poorer prognosis is associated with old age, non-white race, occurrence with an underlying malignancy, bulbar involvement, cardio-respiratory involvement and delayed treatment [6].

An incidence of 5–10 cases per 100,000 individuals has been reported, affecting women more commonly with a ratio of 2:1. Adults and children are equally affected, with the peak incidence between the ages of 45 and 64 in adults. Clinical features include fatigue, proximal muscle weakness and muscle tenderness. Dysphonia, dysphagia and breathlessness can all occur if the respective muscle groups are affected. Extra-muscular manifestations include fever, Raynaud's phenomenon, subcutaneous calcifications and arthralgia. Subcutaneous calcifications are rare in adults [5]. The characteristic rash, previously described, can occur over the shoulders, upper arm and back resulting in the termed "shawl sign". Investigations should include full blood count, urea and electrolytes, liver function tests (transaminases may be raised), creatine kinase (CK) and a chest radiograph. An ECG, echocardiogram and pulmonary function tests should be performed if cardio-respiratory involvement is suspected. Anti-nuclear antibodies (ANA) are positive in 80% of patients, whilst anti Jo-1 antibodies are positive in approximately 20% [5]. CK levels are used to monitor disease activity but levels may have poor correlation with clinical disability [6]. It is imperative that patients are screened for an occult malignancy with investigations that include computed tomography of the abdomen, chest and pelvis, mammography and serum tumour markers. MRI has been used to successfully to identify myositis of affected muscle groups, as muscle oedema correlates well with inflammatory changes. A muscle biopsy is still required to make the diagnosis. Classical findings include endomysial inflammation predominant in the perivascular regions or in the interfascicular septae, and around the fascicles [3].

Principal treatment for DM is corticosteroids. Steroid resistant disease is not uncommon, and methotrexate, azathioprine, cyclosporine and cyclophosphamide have all been used successfully. However a Cochrane Review 2005 [7] demonstrated a lack of high quality randomised controlled trials that have assessed the efficacy and toxicity of immunosuppression in the inflammatory myopathies. Intravenous Immunoglobulin (IV IG) has been used in patients refractory to steroid sparing treatment. The mechanisms of action are not clear but it is known to block the Fc receptors on the vascular wall [4] thus preventing the membrane attack complex deposits from entering endomysial capillaries. IV IG has been to shown to have positive effect on myopathy [3] and on cutaneous ulceration [8]. The dose recommended in the literature is 2 g/kg, to be repeated in six weeks depending upon clinical response. Side effects are mild and include nausea and headaches.

Dysphagia occurs in 10–73% of patients with an inflammatory myopathy [9]. The skeletal muscle activated- oropharyngeal phase of swallowing is clearly affected leading to the increased incidence of aspiration pneumonia [2]. Horowitz et al [10] showed a high incidence of gastric and oesophageal motor dysfunction particularly in DM and PM, therefore suggesting that smooth muscle is also affected. This leads to ineffective peristalsis and clinical manifestation with gastro-oesophageal reflux, gastroparesis and constipation due to colonic inertia [11]. A multi-disciplinary approach is essential, with the roles of dieticians and speech and language therapists being imperative. Assessment with video-fluoroscopic studies can help identify pharyngeal pooling, impaired tongue base retraction, decreased laryngeal elevation and cricopharyngeal dysfunction. Oesophageal and gastric radionuclide transit studies are a safe, simple, non invasive method of highlighting oesophageal and gastric delay. Treatment options include botulinum injection, cricopharyneal dilatation and myotomy. Swallowing compensation techniques also have an important role to play. PEG tube insertion may be required if swallowing remains unsafe but the highest mortality has been described in these patients [2]. Conventional immunosuppressive treatment is recommended but the treatment of choice is IV IG [12].

## Conclusion

Dysphagia commonly occurs in DM and can occur due to oropharyngeal skeletal muscle or distal oesophageal and gastric smooth muscle involvement. Malignancy needs to be excluded. Investigations should include OGD, video fluoroscopy and oesophageal and gastric transit studies. The treatment of choice is IV IG.

## Competing interests

The authors declare that they have no competing interests.

## Consent

Written consent was obtained from the patient prior to submission.

## Authors' contributions

DJ and RM involved in writing of the case report. PW and PK were involved in the review and re-writing of the case report.
